# Phenytoin should not be retired: cost-effectiveness, efficacy, and experience*

**DOI:** 10.1055/s-0046-1817049

**Published:** 2026-03-23

**Authors:** Ana Paula Gonçalves

**Affiliations:** 1 Universidade Federal de Minas Gerais, Faculdade de Medicina, Hospital das Clínicas, Serviço de Neurologia, Belo Horizonte MG, Brazil.; 2 Hospital Felício Rocho, Núcleo Avançado do Tratamento das Epilepsias, Belo Horizonte MG, Brazil.

**Keywords:** Status Epilepticus, Phenytoin, Levetiracetam

## Abstract

Status epilepticus (SE) is a common neurological emergency that occurs in children and adults. It can be treated with different antiseizure medications (ASMs). Benzodiazepines represent the first line of treatment. If seizure activity does not cease, it is a common clinical practice to prescribe the patient either phenytoin (PHT), levetiracetam, or valproate. For decades, PHT has remained a cornerstone in the treatment of certain types of seizures, despite the emergence of newer agents such as levetiracetam. The current article explores the ongoing relevance of phenytoin, its unique advantages over levetiracetam in specific contexts, and the importance of individualized treatment in epilepsy management.

Status epilepticus (SE) is a common neurological emergency that occurs in children and adults, and it may be a serious condition that can affect vital and functional prognosis and requires urgent treatment. 1 2 The current definition of SE, as stated by the International League Against Epilepsy (ILAE), is “a condition resulting either from the failure of the mechanisms responsible for seizure termination or from the initiation of mechanisms, which lead to abnormally-prolonged seizures (after time point T1). This condition can have long-term consequences (after time point T2), including neuronal death, neuronal injury, and alteration of neuronal networks, depending on the type and duration of seizures”. 3

Since the historic Veterans Affairs Status Epilepticus study, 4 it has been well established that benzodiazepines are the first-choice antiseizure medications (ASMs) for the treatment of convulsive SE. If benzodiazepines do not stop the seizures, the next medication to administer remains under debate. 5 Despite the availability of newer ASMs, phenytoin (PHT) is still used for its efficacy, cost-effectiveness, and the established experience of healthcare providers in its use. Moreover, it is particularly beneficial in specific patient populations, such as those with SE or those who have had suboptimal responses to newer therapies. 6 7 8

Three major randomized trials to address the question of the treatment of established SE (defined by benzodiazepine failure) were published in 2019: 9 The Emergency Treatment with Levetiracetam or Phenytoin in Status Epilepticus in Children 10 (EcLiPSE) and the Levetiracetam versus phenytoin for second-line treatment of convulsive status epilepticus in children 11 (ConSEPT) were pediatric, open-label, multicenter studies, and the Established Status Epilepticus Treatment Trial 12 (ESETT) was a large, long-awaited, multicenter study in which valproate, levetiracetam (LVT) and fosphenytoin were compared in children and adults with SE at 57 centers in the United States. These studies have demonstrated that LVT is a viable alternative to PHT; however, they showed no advantages for LVT compared with PHT. 9

Since 1968, parenteral PHT has become the first choice for the treatment of SE after failure of benzodiazepine therapy. Intravenous PHT was introduced in 1956, and its first use in SE was reported in 1958. However, its clinical value was not realized until 1968, when Wallis et al. published a pioneering study involving 31 patients with SE who were treated with PHT at a loading dose of 1,000 mg. 13

To appreciate the comparative roles of PHT and LVT, it is essential to understand their mechanisms of action and established clinical profiles. Phenytoin primarily exerts its anticonvulsant effects by modulating voltage-gated sodium channels, thereby stabilizing neuronal membranes and inhibiting the repetitive firing of action potentials. It also influences calcium channels and enhances Gamma-aminobutyric acid (GABA)ergic inhibition, contributing to its broad-spectrum antiepileptic activity. Phenytoin has been a first-line treatment for various seizure types for decades, particularly focal seizures and generalized tonic-clonic seizures. Its efficacy in these areas is well-established through extensive clinical experience and numerous studies. 14 15 Levetiracetam, on the other hand, operates through a distinct mechanism, primarily by binding to synaptic vesicle protein 2A (SV2A). This interaction is thought to modulate neurotransmitter release, thereby reducing neuronal excitability. Levetiracetam has a broad spectrum of activity, and it is effective against focal seizures, myoclonic seizures, and generalized tonic-clonic seizures. 16

According to a large systematic review with meta-analysis published in 2020, 17 LVT was not superior to PHT in the primary outcome of seizure cessation, nor in secondary outcomes such as seizure recurrence and adverse effects, except for cardiac arrhythmia. Despite its better pharmacokinetic profile, when compared head to head, LVT was not shown to be better tolerated or to result in a lower incidence of adverse effects. The frequency of significant side effects was not significantly higher in the fosphenytoin group 4 (3.2%) of life-threatening hypotension, zero life-threatening cardiac arrhythmia, compared with the LVT group, 1 (0.7%) hypotension, 1 (0.7%) cardiac arrhythmia in the ESETT. Furthermore, some etiologies of SE are *supposed* to respond better to medications that act on sodium channels. 9

When considering patients with preexisting psychiatric conditions or those who are particularly sensitive to mood alterations, the potential for LVT to exacerbate these issues might make PHT a more favorable option, provided its other side effects can be managed. The choice often boils down to a personalized risk-benefit assessment. 18

While LVT boasts a broad spectrum of efficacy, PHT retains a strong position for specific seizure types and patient characteristics. Phenytoin has a particularly robust track record in the management of focal seizures and generalized tonic-clonic seizures. For these seizure types, PHT often provides excellent control, and for patients who tolerate it well, it can be a highly efficacious therapy 14 15

One of the most compelling arguments for the continued use of PHT, especially in resource-limited settings, is its cost-effectiveness. Phenytoin is an older generic drug; as such, its acquisition cost is significantly lower than that of newer ASMs such as LVT. A cost-effectiveness analysis 19 comparing PHT and LVT for early seizure pharmacoprophylaxis after traumatic brain injury found that PHT was a more cost-effective option. The affordability of PHT enables broader access to effective seizure control, making it a critical choice for many patients globally.

While LVT offers distinct advantages, a critical examination reveals that PHT still has a valid and, in certain aspects, even superior role in the management of SE.

It is crucial to acknowledge that PHT remains a highly-effective option, particularly when rapid intravenous access is established and appropriate cardiac monitoring is in place. Its long-standing use and familiarity among clinicians contribute to its continued relevance in emergency settings.

In conclusion, while newer ASMs such as LVT have certainly expanded the therapeutic arsenal for epilepsy, it would be premature and clinically unwise to relegate PHT to the annals of medical history. Phenytoin continues to have a valid and, in certain aspects, superior role in modern epilepsy management.

Its proven efficacy in specific seizure types, particularly focal and generalized tonic-clonic seizures, remains robust. Crucially, its significant cost-effectiveness makes it an indispensable option globally, ensuring access to vital seizure control for a broader population. Furthermore, while LVT's behavioral side effects can be a considerable concern for some patients, PHT's known adverse-effect profile is predictable and manageable with careful monitoring, offering a distinct advantage for those susceptible to psychiatric disturbances.

The decision to use PHT or LVT, or any ASM, must be individualized, taking into account seizure type, patient comorbidities, potential side effects, pharmacokinetic interactions with other medications, and socioeconomic factors. For many patients, PHT remains a powerful, effective, and economically-viable choice, demonstrating that its enduring legacy in epileptology is far from over.
